# Knowledge, Attitude, and Practice on Salt and Assessment of Dietary Salt and Fat Intake among University of Sharjah Students

**DOI:** 10.3390/nu11050941

**Published:** 2019-04-26

**Authors:** Leila Cheikh Ismail, Mona Hashim, Amjad H. Jarrar, Maysm N. Mohamad, Sheima T. Saleh, Nada Jawish, Mayssaa Bekdache, Hiba Albaghli, Dyana Kdsi, Dina Aldarweesh, Ayesha S. Al Dhaheri

**Affiliations:** 1 Clinical Nutrition and Dietetics Department, College of Health Sciences, University of Sharjah, Sharjah 27272, United Arab Emirates; mhashim@sharjah.ac.ae (M.H.); U14120207@sharjah.ac.ae (S.T.S.); U14123049@sharjah.ac.ae (N.J.); U14121578@sharjah.ac.ae (M.B.); U00025698@sharjah.ac.ae (H.A.); U14121165@sharjah.ac.ae (D.K.); U14123562@sharjah.ac.ae (D.A.); 2 Nutrition and Health Department, College of Food and Agriculture, United Arab Emirates University, Al Ain 15551, United Arab Emirates; amjadj@uaeu.ac.ae (A.H.J.); maysmnezar88@uaeu.ac.ae (M.N.M.); ayesha_aldhaheri@uaeu.ac.ae (A.S.A.D.)

**Keywords:** dietary salt, dietary fat, students, knowledge, attitude, practice

## Abstract

Background: Cardiovascular diseases are the main cause of deaths in the United Arab Emirates and reducing dietary salt intake is recommended to improve the population’s health. Methods: a cross-sectional survey was given to 401 students from the University of Sharjah to investigate knowledge, attitudes, and practices related to dietary salt intake and a 24-h dietary recall among a subsample of 122 students, to assess the dietary intake of total fat, cholesterol, saturated fat, trans fat, and sodium. Results: findings indicated low salt-related knowledge scores among students (17 out of 30), high prevalence of overweight (28%), obesity (14%), hypertension stage 1 (31%), and hypertension stage 2 (20%). The results also revealed a high percentage of students exceeding the recommended intake of total fat (48%), saturated fat (90%), trans fat (64%), and sodium (89%), and all students not meeting potassium recommendations. Conclusions: culture-specific awareness campaigns on salt and fat intake and their association with health are needed.

## 1. Introduction

Noncommunicable diseases (NCDs) are considered one of the main health challenges of the 21st century as they represent a major risk to human health and economic growth especially in low-income and middle-income countries [1]. The World Health Organization’s (WHO) report on the global status of NCDs in 2014 reported that out of the 56 million deaths that occurred worldwide during 2012, around 38 million deaths were linked to NCDs [2]. Therefore, NCDs are currently causing more deaths than all other causes of morbidity combined and it is projected that NCD deaths will increase to 52 million by 2030 [2]. Moreover, about 46% of NCDs deaths are related to cardiovascular diseases (CVDs) and about 1.7 million annual deaths from CVDs are attributed to excess sodium/salt intake [3]. Additionally, evidence suggests that high blood pressure is one of the major risk factors for the development of CVDs [3,4]. Worldwide salt is the main dietary source of sodium and excess consumption of dietary sodium has been found associated to an increased risk of hypertension and CVDs [3,5,6].

In the United Arab Emirates (UAE), NCDs are estimated to account for 77% of all deaths and of these 40% are caused by CVDs [7]. Reducing salt intake has been identified as one of the most cost effective measures for improving population health [8]. Clinical trials showed that lowering dietary sodium intake can reduce CVDs risk by reducing high blood pressure [8,9,10]. A study in the United States intended to explore the potential impact of reducing dietary salt by 3 g per day estimated a decrease in annual healthcare costs by a range of $10 billion to $24 billion, and projected a significant decrease in the incidence of coronary heart disease, stroke, and myocardial infarction [11]. Hence, the WHO recommends reducing salt intake to less than 5 g/day and sodium to less than 2 g/day in adults to help prevent high blood pressure, coronary heart disease, and stroke [6].

Limiting dietary cholesterol, saturated fat, and trans-fat intakes have also been recommended for people at high risk of, or with, CVDs [12,13,14,15,16]. In 2006, the American Heart Association (AHA) recommended limiting the intake of saturated fat to 7% of energy, trans fat to 1% of energy, and cholesterol to 300 mg/day [16]. The recent AHA guidelines in 2013 suggested aiming for a dietary pattern that achieves 5% to 6% of calories from saturated fat and reducing the percent of calories from trans-fat [13]. Information on dietary cholesterol, saturated fat, and trans-fat intakes in the UAE is scarce. However, several studies have reported dietary changes in the Emirati diet contributing to the high prevalence of CVDs, including the replacement of traditional foods (high in fiber and low in fat, cholesterol, and sodium) with energy-dense fast foods (high in fat, cholesterol, sugars and sodium, and low in dietary fiber) [17,18,19,20].

In 2014, the Gulf Cooperation Council Executive Board of Health Ministers had a meeting to discuss reducing the growing burden of NCDs in the region and proposed a reduction in salt and fat intakes as the main target to meet by 2025 [21]. However, to plan and develop effective interventions to achieve these targets, efforts should initially focus on the assessment of current dietary salt and fat intake among the population. Moreover, the WHO recommends assessing the knowledge, attitudes, and practices toward dietary salt in the planning for any intervention aimed to reduce salt consumption among a population [2,6].

Available literature revealed that the Emirati diet is high in fat, cholesterol, and sodium, and low in dietary fiber [17,18,19,20]; the mean salt intake in the UAE was estimated to be around 9 g/day (about 3.5g /day of sodium) [7]. Considering these facts, highlighting the importance of assessing salt and fat intake and determining salt- and fat-related knowledge, attitudes, and practices to assist in the development of national salt and fat reduction interventions.

The current study is a cross-sectional survey study among the University of Sharjah (UOS) students, which aims to (1) investigate knowledge, attitudes and practices (KAP) related to dietary salt intake among UOS students aged 18–45 years, and (2) assess the dietary intake of total fat, cholesterol, saturated fat, trans fat, and sodium using 24-h dietary recall among a subsample of the students.

## 2. Materials and Methods 

### 2.1. Study Design and Subjects

This is a cross-sectional survey of students at the University of Sharjah (UOS) in the city of Sharjah, United Arab Emirates. It was conducted during the academic year 2017/2018. The sample size was calculated based on the following formula to be representative of all students enrolled in UOS; with a confidence interval of 95%.

N = z^2^ × P × (1 − P)/e^2^

z = level of confidence (for a level of confidence of 95%, z = 1.96); P = estimated proportion of the population that presents the characteristic P = 0.5; e = margin of error, e = 0.05; N (sample size) = 384 participants, plus 20% = approximately 461 participants.

The sampling method was a stratified random sampling. All university students were divided into strata by college, then a random subsample consisting of 5% of the students from each college was selected. Those were then contacted through e-mail to voluntarily participate in the study. A sample of 544 received an invitation to participate in the study, which included 37 students from each college. Only 401 students showed interest and participated in the survey, thus the response rate was 74%.

A self-administered questionnaire was administered by senior students majoring in Clinical Nutrition and Dietetics between February and April 2018. Inclusion criteria included: students with no history of hypertension and were not taking diuretics or hypertension medications. Data collection took place at the nutrition clinic during week days at different times of the day to guarantee student’s ability to participate. Each participant was given an information sheet explaining the purpose and nature of the study. Participants were free to withdraw at any time. A written informed consent to participate in the study was obtained from all participants before taking part. Consenting participants (*n* = 401) were then asked to fill a self-administered questionnaire and a sub-sample of the participants (*n* = 122) were interviewed to obtain a 24-h food recall for one day. An identification number was assigned to each participant to maintain anonymity.

The study obtained ethical approval from the University of Sharjah Research Ethics Committee (UOS-REC) reference number: REC-18-03-09-01-S.

### 2.2. Study Instrument

Anthropometric measures, including height and weight, were assessed in the nutrition clinic of the Clinical Nutrition and Dietetics department. Body mass index (BMI) was calculated by dividing the weight in kilograms by the height squared in meters (kg/m^2^). The WHO’s guideline for the classification of adults according to BMI was used to categorize participants into groups [22]. Systolic and diastolic blood pressures were measured in triplicate after five minutes of resting in a seated position using a calibrated digital automated blood pressure monitor (Omron HEM-907-E7). The readings were repeated at three-minute intervals and the mean of the last two readings was recorded. Participants were designated as hypertensive if their systolic BP was ≥130 mmHg and/or diastolic BP ≥ 80 mmHg [23].

A 24-h food recall was obtained from a sub-sample of the participants (*n* = 122) to assess the intake of total fat, cholesterol, saturated fat, trans fat, and sodium. The researchers interviewed each participant and used visual aids to facilitate serving size estimations. Essential information such as timing, location of meals, methods of food preparation, sources of food, table salt consumption, and portion sizes were collected. Food Processor Nutrition Analysis Software-ESHA (version 10.4; ESHA Research, Salem, OR, USA) was used to estimate the energy and nutrient content of the consumed foods.

A multicomponent KAP (knowledge, attitudes, and practices) survey model was adapted [24] and the questions were modified based on past surveys used in similar studies [25,26,27,28]. The first part of the questionnaire included sociodemographic questions (age, gender, college, nationality, residence, and marital status). The rest of the questionnaire was divided into three main sections: knowledge, attitudes, and practices related to salt consumption.

#### 2.2.1. Knowledge

The knowledge part of the questionnaire inquired about the percentage of sodium in salt (response options “20%”, “40%”, “60%”, “I don’t know”), the relationship between high salt intake and specific health conditions such as hypertension, cardiovascular disease, fluid retention, fever, renal diseases and diabetes, and if participants think that reducing salt intake improves their blood pressure and overall health (response options “yes”, “no”, “I don’t know”). Participants were also asked to classify specific foods as high or low in salt/sodium [29].

#### 2.2.2. Attitude

Five questions were asked to assess participant’s attitude towards salt/sodium. Three of them inquired about how important it is for the participant to reduce the amount of salt he/she adds to foods, his/her overall sodium intake, and the amount of processed food he/she consumes. Response options were “disagree”, “neutral” and “agree”. The fourth question inquired if the participant is concerned about the amount of salt/sodium in his/her diet (response options “yes”, “no”) and the last question was about the amount of salt he/she thinks they consume (response options “too much”,” just the right amount”, “far too little”).

#### 2.2.3. Practice

The practice part of the questionnaire inquired about the use of food labels, if the information on food labels affects purchasing decisions, if participants check labels specifically for salt/sodium content, whether salt/sodium content indicated on the label affects purchasing decisions, and if participants try to buy “low salt” or “no added salt” food products. In addition, practice related to the daily use of salt was assessed by five questions inquiring about the frequency of adding salt or stock cubes during cooking and whether he/she adds salt to food prior to tasting or at the table. Responses for practice related questions included “never”, “sometimes” and “often”. Two additional questions queried whether participants tried to reduce salt intake before and if spices were used as an alternative to reduce salt addition (response options “yes”, “no”, “I don’t know’). The final question asked about the type of bottled water commonly consumed by the participants (response options “regular filtered water” (Masafi, Al Ain, Arwa, etc.), “low sodium filtered water”, “I don’t know”).

### 2.3. Data Analysis

Data was analyzed using SPSS software, version 25.0 (SPSS, Chicago, IL, UASA). Continuous data were expressed as mean ± standard deviation and categorical data were expressed as counts and percentages. A knowledge score ranging from 0 to 30 was calculated for each participant based on the number of correct answers to knowledge related questions. Likewise, an attitude score ranging from 0 to 5 was calculated for each participant based on the number of favorable attitude related statements. Additionally, a practice score ranging from 0 to 13 was calculated for each participant based on the number of favorable practice related statements. One-way ANOVA and Student t-test were used to compare groups. Pearson’s chi-square test was used to determine the association between gender and proportions of knowledge, attitude, and practice rates. Independent samples t-test was conducted on dietary intake measure to assess between gender differences. Lastly, *p* values < 0.05 were considered statistically significant.

## 3. Results

### 3.1. Sample Charactaristics

A sample of 544 students were invited to participate in the study, but only 401 students (52.1% males; 47.9% females) agreed to participate (response rate 74%).

As shown in Table 1, participants’ age ranged between 18 and 25 years. The female to male ratio was almost 1:1, with 52.1% males. The majority of participants (89%) were non-Emirati students, (64.1%) were living with their family, and (97.8%) were single. About 57% of students were specializing in health-related majors. More than one third of the study sample were classified as overweight and obese (28.2% and 13.5%, respectively, based on their BMI). Although only participants reporting not being previously diagnosed with hypertension were included in this study, about half (51.1%) of the participants were found to have hypertensive stage 1 and hypertensive stage 2 when their blood pressure was measured.

### 3.2. Salt-Related Knowledge, Attitudes, and Practices in the Study Population

Only 35.5% of study participants were able to report the correct percentage of sodium in salt (Table 2). However, the majority of the study participants were able to correctly describe the relationship between high salt intake and hypertension, cardiovascular diseases, water retention, and renal diseases (70.3%, 65.6%, 62.6%, and 64.6%, respectively). Furthermore, over 84% of study participants recognized that reducing salt intake would help improve both overall health status and blood pressure. The results also showed that less than one third of the participants were able to correctly identify pita bread, Iranian bread, and corn flakes as a high source of salt food items (23.4%, 30.7%, and 30.4%, respectively). On the other hand, around 70% of the participants correctly categorized cheddar cheese, pickles, chicken cubes, and instant noodles as high in sodium food items. Gender differences in salt-related knowledge were noted especially when identifying sodium content in foods. Women had a significantly higher percentage in providing correct answers to the sodium content in several food items such as canned vegetables, cheddar cheese, pickles, salad dressing oil, ketchup, tomato paste, chicken cubes, instant noodles, and filtered water (*p* < 0.001).

Table 3 presents salt-related attitudes of participants (*n* = 401) according to gender. Over one half of participants (57.1%) believed that they were consuming just the right amount of salt on a daily basis. However, only 12.7% of participants stated that they were concerned about the amount of salt in their diet, with a significantly high percentage of concerned females (30%, *p*-value = 0.012). Similarly, only 21.2% of participants agreed that reducing added salt to foods is important to them. This is justifiable, since the majority of participants believe that they consume just the right amount of salt, thus there is no need to reduce their current intakes.

Salt-related practices among participants by gender are presented in Table 4. Only 17.2% of participants stated that they often check food labels and 20.4% reported that the information on food labels affects their purchasing decisions. Moreover, an even lesser percentage of participants reported specifically checking for salt content on the food label (5.7%) and that their food choices are affected by the salt/sodium content (6.5%). Similarly, only 9.2% and 8.2% of participants stated that they often try to buy “low salt” or “no added salt” foods, respectively. On the other hand, 61.1% of students often add salt to food while cooking, 15.2% often use stock cubes during cooking, 25.9% often add salt to food at the table, and 14.2% often add salt to food even before tasting it. Gender differences in relation to salt related practices were only significant as more females reported trying to reduce salt by using spices instead (*p*-value = 0.005).

Knowledge, attitude, and practice scores were derived for each participant based on the number of correct/favorable answers provided in each section of the survey. Knowledge scores can range between 0 and 30; the higher the score the higher the knowledge. Attitude scores can range between 0 and 5; the higher the score the more positive is the attitude towards reducing salt intake. Practice scores can range between 0 and 13; the higher the score the more positive is the practice towards reducing salt intake.

Table 5 shows that belonging to a higher age group (21–25 years) was associated with a significant increase in knowledge scores (*p* = 0.010). Moreover, females had a significantly higher knowledge scores compared to males (*p* < 0.001). Similarly, having specialized in a health-related major was significantly associated with a higher knowledge score (*p* = 0.009). The results also showed that students who reported consuming most meals at restaurants had a significantly lower attitude and practice scores (*p* < 0.001)

### 3.3. Assessement of Dietary Intake among UOS Students

Table 6 presents food intake of macronutrients among UOS students (*n* = 122) according to gender. The results showed that protein intake was significantly higher among males (92.1 ± 41.0) compared to females (72.1 ± 43.5) (*p*-value = 0.011). However, the percentage of energy from protein was within the Acceptable Macronutrient Distribution Rang (AMDR) for both genders (within 10–35%) [30]. The percentage of energy from total carbohydrates was significantly higher among females (49.6 ± 10.3) compared to males (43.7 ± 13.8) (*p*-value = 0.007), yet within AMDR for both genders (45–65%) [30]. Total fat intake was slightly higher than the AMDR for fat (20–35%) among males. Likewise, males reported consuming a significantly higher amount of saturated fat, monounsaturated fatty acids, polyunsaturated fatty acids, trans fat, and cholesterol (*p*-values = 0.012, 0.001, 0.004, 0.024, and <0.001, respectively). The ratio of poly- to monounsaturated fat was almost 0.5. Sodium intake was 3677 mg/day among males and 3464 mg/day among females, which was much higher than the recommendations of the Institute of Medicine (IOM) of the National Academies (<2300 mg/day) [30]. Furthermore, potassium intake for both genders was lower than the recommended adequate intakes value (AI = 4700 mg/day) by about 3000 mg/day [31].

Table 7presents the percentage of UOS students who did not meet the dietary recommendations of the AHA and the IOM (*n* = 122) according to gender [16,30,31]. The results show no significant differences in the proportion of male and female students who failed to meet the recommendations, except for cholesterol intake (*p*-value < 0.001) as females scored better. Moreover, 55.4% of males and 42.4% of females consumed high dietary fat intake (>35%). Also, 91.1% of males and 89.4% of females exceeded 7% of total calorie intake from saturated fatty acids. Approximately, 89% of students failed to meet sodium recommendations (>2300 mg/day) and all students did not meet the recommended adequate intakes value of potassium (AI = 4700 mg/day).

## 4. Discussion

This study has investigated salt-related knowledge and attitudes and the impact of these perceptions on salt-related practices among a sample of university students in Sharjah, UAE. Additionally, a one day 24-h dietary recall was collected from a sub-sample of students to assess the dietary intake of total fat, cholesterol, saturated fat, trans fat, and sodium.

Results from the questionnaire pointed out that university students’ knowledge related to salt and sodium was relatively low, and a lesser percentage of students considered implementing practices to reduce salt/sodium in their diet. This might be due to believing that they consume just the right amount of salt per day as reported in the attitude section of the questionnaire. Moreover, the majority of students were aware of the adverse health effects associated with high salt intake. The study’s findings are comparable to a study conducted in Lebanon examining salt-related knowledge, attitude, and behaviors among adult Lebanese consumers [25]. Consumers in the Lebanese study were able to identify excessive salt consumption as a risk factor for high blood pressure [25].

The findings of the study showed that most students had limited knowledge related to the main foods that contribute salt in the diet, such as pita bread, Iranian bread, and corn flakes as a high source of salt food items. Although, cereal products, in particular bread (a popular staple food in Arab countries), was found to be the major contributor of salt in the Gulf diet [21]. This suggests that students in the UAE are unaware of some of the main sources of dietary salt in their diet. These findings are in line with those reported by previous Western studies investigating attitudes, knowledge, and behavior related to salt consumption [26,27,32].

On the other hand, in Kuwait, a close Gulf country, it was indicated that bread consumption was the second main source of salt after Kuwaiti composite dishes prepared at home [33]. Similarly, in Qatar the government found that the main source of salt in the Qatari diet was from bread and other baked products [21]. Furthermore, data from food composition tables in the Middle East demonstrated high sodium content in the diet of Middle Easterners, due to the high use of table salt, spices, and pickles [34,35,36]. Therefore, nutritional education focusing on the healthy preparation of food at home, using less salt and fat while cooking, and limiting consumption of high salt foods is recommended. Fortunately, a reduction of the salt level in bread ranging from 20% to 30% reduction is currently implemented in all Gulf countries as an effective means to reduce the NCDs burden in the region [21]. A recent systematic review and meta-analysis determining consumer acceptance of reformulated food products showed that salt can be reduced by about 40% in breads and approximately 70% in processed meats without significantly impacting consumer acceptability [37]. Furthermore, a study assessing consumer acceptance of reductions in calories, fat, saturated fat, and sodium to restaurant recipes, showed that consumer acceptable modifications to menu items were up to 210 calories, 20 g fat, 8 g saturated fat (≤26% of calories), and 1970 mg sodium (≤31% sodium) per serving [38]. This kind of approach facilitates the selection of healthier choices by the consumer.

The results showed that less than a quarter of participants check food labels and use the information on food labels to guide their purchasing decisions. More alarmingly, less than 10% check for salt content on the food label and use it to guide their food choices. These findings are much lower than those reported in Lebanon and Australia [25,39], which requires special attention to the fact that salt is not perceived as a major health risk among students and food labels are not utilized by students. Therefore, awareness of the health risks associated with high salt consumption is recommended to increase salt label usage and purchases of low salt foods. In addition, awareness programs on the importance of reading nutrition and salt labels to guide purchasing decisions should be regularly implemented at schools and universities. It is also recommended to establish a consumer-friendly nutrition labeling system that includes salt on the front of the pack or uses traffic light labeling as these labels were found associated with fewer unhealthy purchases [40,41].

The results of this study identified older age, female gender, and studying health related majors as characteristics associated with higher salt-related knowledge scores. Moreover, students who reported consuming most of their meals at restaurants were more likely to adopt less favorable salt-related attitudes and practices. These findings are in line with other studies reporting older participants having higher levels of nutrition knowledge and females being more health-conscious, thus having higher salt related knowledge scores compared to males [25,39,42].

The results of the 24-h recall presented a high percentage of students exceeding the recommendations of total fat, saturated fat, trans fat, and sodium (48%, 90%, 64%, and 89%, respectively), and all students did not meet the recommended intake of potassium. This is in line with previous studies in the UAE indicating high intake of fast foods and low intake of fruits and vegetables [17,18,19,20]. Similarly, a study on Omani adolescents, a neighboring Gulf country, reported that more than 50% of studied participants consumed fast food more than four times per week, however, less than 15% of participants consumed fruits and vegetables more than four times per week [43]. Likewise, a study among students at the University of Valencia found that students were consuming a diet high in protein, fat, and saturated fat [44].

It is worth to note that low potassium intake accompanied with high sodium intake among university students was related substantially to the little consumption of fruits and vegetables. It is well documented that an appropriate intake of potassium is recommended as it opposes the negative effects of sodium chloride [45]. Besides, it is well established that high salt intake has been identified as a risk factor for hypertension and elevated blood pressure, and apparently our sample demonstrated this finding as half (51.1%) of the participants’ blood pressure readings was categorized as hypertensive stage 1 and hypertensive stage 2. The higher prevalence reported in this study in comparison to studies from the region [46] is because the recent definition for hypertension by the American College of Cardiology and American Heart Association (ACC/AHA, 2017) [23] was used instead of the previous one provided in the seventh report of the joint national committee on prevention, detection, evaluation, and treatment of high blood pressure (JNC7, 2003) [47].

Further the high fat intake finding encountered among the university students should be critically considered in a country witnessing a 2–3-fold increase in the prevalence of overweight and obesity between 1989 and 2017, as detected in a recent systematic review [48]. The review also noted a high prevalence of cardiometabolic disorders in the UAE. Consistently our sample had an alarming amount of hypertensive cases which were not diagnosed by the students upon inclusion. These results collectively require the need for intervention strategies that are attractive for the young generation using social media which has greater exposure to “fashionable” foods and is more vulnerable to emerging marketing trends [49]. A variety of awareness campaigns utilizing the university’s social media platforms could be employed to promote incorporating fruits and vegetables as healthy food choices among university students. In addition, planning for activities such as cooking competitions in conjunction with local restaurants for preparing heart-healthy meals in line with student preferences or the traditional dietary habits.

It is acknowledged that this study has a number of limitations. This study was restricted to university students; therefore, the findings may not be extrapolated to all young adults in the UAE. In addition, the questionnaire was based on self-reported attitudes and practices which may not accurately reflect actual attitudes and practices. The use of 24-h dietary recall has several limitations, such as systematic errors, respondent bias, under or over-reporting, interviewer biases, and incorrect estimation of portion size [50]. However, the objective of the study was to provide an approximate estimation of dietary intake among university students.

Despite the limitations of the study, the findings presented in this paper highlight the need to increase awareness regarding salt recommendations for different age groups, identify main sources of sodium in the Emirati diet, work with food manufacturers to progressively reduce sodium/salt in their products to meet recommendations, and use media to highlight the health benefits of reducing salt consumption and choosing low salt products. Collaboration between health authorities and the Ministry of Education to include sufficient information on preventative measures against chronic diseases in school and universities curriculums is highly recommended.

## 5. Conclusions

This study provided a better understanding of salt-related knowledge, attitude, and practices among a sample of university students, and revealed their recommended intakes of total fat, saturated fat, trans fat, and sodium, thus highlighting the need to develop culture-specific awareness campaigns on salt and fat intake, and their association with health. It is hopeful that this study may provide insight into future studies addressing the young generation’s barriers to salt and fat reduction, and support current efforts aimed to reduce salt and fat in the Gulf region taking into consideration that taste and acceptance are not compromised.

## Figures and Tables

**Table 1:** Socio-demographic and physical characteristics of study participants (*n* = 401).

Characteristics	*n*	(%)
Age (years)		
18–20	198	(49.4)
21–25	203	(50.6)
Gender		
Male	209	(52.1)
Female	192	(47.9)
Nationality		
Emirati	44	(11.0)
Arab ^2^	347	(86.5)
East Asian	8	(2.0)
Westerner	2	(0.5)
Non-Emirati	357	(89.0)
Health related major ^1^		
Yes	230	(57.4)
No	171	(42.6)
Residential type		
With Family	257	(64.1)
Hostel	133	(33.2)
Alone	11	(2.7)
Marital status		
Single	392	(97.8)
Married	9	(2.2)
Most meal are consumed at		
Home	246	(61.3)
Restaurants	155	(38.6)
Body Mass Index (kg/m^2^)		
Underweight (<18.5)	10	(2.5)
Normal weight (18.5–24.99)	224	(55.9)
Overweight (25–29.99)	113	(28.2)
Obesity (>30)	54	(13.5)
Blood Pressure (mm Hg)		
Normal (< 120/80)	153	(38.2)
Elevated (systolic 120–129 and diastolic <80)	43	(10.4)
HBP ^3^ Stage 1 (systolic 130–139 or diastolic 80–89)	126	(31.4)
HBP Stage 2 (systolic >140 or diastolic ≥90)	79	(19.7)

^1^ Health-related major includes health sciences, medicine, dental medicine, and pharmacy; ^2^ from the Middle East and other Gulf countries; ^3^ HBP: high blood pressure.

**Table 2:** The number and percentage of participants who answered knowledge related questions correctly among University of Sharjah (UOS) students (*n* = 401) by gender.

Variable ^1^	Total *n* (%)	Males *n* (%)	Females *n* (%)	*p*-Value
Percentage of sodium in salt (40%)	142 (35.5%)	74 (35.4%)	142 (35.5%)	0.499
High salt intake may increase risk factors for				
Hypertension (Yes)	282 (70.3%)	144 (68.9%)	138 (71.9%)	0.117
Cardiovascular diseases (Yes)	263 (65.6%)	135 (64.6%)	128 (66.7%)	0.397
Diabetes (No)	213 (30.2%)	119 (56.9%)	94 (49.0%)	0.03
Fever (No)	200 (49.9%)	101 (48.3%)	99 (51.6%)	0.55
Water retention (Yes)	251 (62.6%)	122 (58.4)	129 (67.2%)	0.092
Renal diseases (Yes)	259 (64.6%)	126 (60.3%)	133 (69.3%)	0.113
Reducing salt intake will improve				
Health (Yes)	339 (84.5%)	164 (78.5%)	175 (91.1%)	0.002
Blood Pressure (Yes)	348 (86.8%)	173 (82.8%)	175 (91.1%)	0.041
Sodium content in the following foods is				
Pita bread (High)	94 (23.4%)	42 (20.1%)	52 (27.1%)	0.145
Iranian bread (High)	123 (30.7%)	61 (29.2%)	62 (32.3%)	0.358
Fruits (Low)	298 (74.3%)	147 (70.3%)	151 (78.6%)	0.16
Fresh vegetables (Low)	296 (73.8%)	144 (68.9%)	152 (51.4%)	0.065
Frozen vegetables (Low)	178 (44.4%)	90 (43.1%)	88 (45.8%)	0.431
Canned vegetables (High)	245 (61.1%)	106 (50.7%)	139 (72.4%)	<0.001
Cheddar cheese (High)	292 (72.8%)	132 (63.2%)	160 (83.3%)	<0.001
Pickles (High)	294 (73.3%)	137 (65.6%)	157 (81.8%)	0.001
Olive oil (Low)	208 (51.9%)	104 (49.8%)	104 (54.2%)	0.254
Basmati rice (Low)	184 (45.9%)	89 (42.6%)	95 (49.5%)	0.381
Egyptian rice (Low)	148 (36.9%)	77 (36.8%)	71 (37.0%)	0.75
Milk, yoghurt (Low)	208 (51.9%)	103 (49.3%)	105 (54.7%)	0.284
Salad dressing oil (High)	252 (62.8%)	117 (46.4%)	135 (70.3%)	0.001
Ketchup (High)	277 (69.1%)	125 (59.8%)	152 (79.2%)	<0.001
Tomato paste (High)	253 (63.1%)	110 (52.6%)	143 (74.5%)	<0.001
Red meat (Low)	131 (32.7%)	75 (35.9%)	56 (29.2%)	0.171
Poultry (Low)	153 (38.2%)	71 (34.0%)	82 (42.7%)	0.148
Corn flakes (High)	122 (30.4%)	59 (28.2%)	63 (32.8%)	0.33
Chicken cubes (High)	275 (68.6%)	125 (59.8%)	150 (54.5%)	<0.001
Instant noodle (High)	283 (70.6%)	128 (61.2%)	155 (80.7%)	<0.001
Filtered water (Low)	274 (68.3%)	131 (62.7%)	143 (74.5%)	0.009

^1^ The correct answers are provided in brackets next to each variable.

**Table 3:** Salt-related attitudes among UOS students (*n* = 401) by gender.

Variable ^1^	Total *n* (%)	Males *n* (%)	Females *n* (%)	*p*-Value
How much salt do you think you consume (Just the right amount)	229 (57.1%)	119 (56.9%)	110 (57.3%)	0.126
Are you concerned about the amount of salt/sodium in the diet (Yes)	51 (12.7%)	21 (10.0%)	30 (15.6%)	0.012
Reducing added salt to foods is important to you (Agree)	85 (21.2%)	42 (20.1%)	43 (22.4%)	0.544
Reducing consumption of processed foods is important to you (Agree)	127 (31.7%)	71 (34.0%)	56 (29.2%)	0.197
Reducing your sodium intake is important to you (Agree)	84 (20.9%)	36 (17.2%)	48 (25.0%)	0.161

^1^ Attitude was assessed based on a three-point Likert scale but only answers of “agree” are presented.

**Table 4:** Salt-related practices among UOS students (*n* = 401) by gender.

Variable ^1^	Total *n* (%)	Males *n* (%)	Females *n* (%)	*p*-Value
Check food labels (Often)	69 (17.2%)	30 (14.4%)	39 (20.3%)	0.119
Information on food labels affects purchasing decisions (Often)	82 (20.4%)	40 (19.1%)	42 (21.9%)	0.405
Check labels specifically for salt/sodium content (Often)	23 (5.7%)	9 (4.3%)	14 (7.3%)	0.344
Salt/sodium content on label affects purchasing decisions (Often)	26 (6.5%)	12 (5.7%)	14 (7.3%)	0.344
Try to buy “low salt” foods (Often)	37 (9.2%)	14 (6.7%)	23 (12.0%)	0.180
Try to buy “no added salt” foods (Often)	33 (8.2%)	15 (7.2%)	18 (9.4%)	0.718
Add salt to food during cooking (Often)	245 (61.1%)	130 (62.2%)	115 (59.9%)	0.617
Use Stock Cubes during cooking (Often)	61 (15.2%)	27 (12.9%)	34 (17.7%)	0.323
Add salt to food at the table (Often)	104 (25.9%)	52 (24.9%)	52 (27.1%)	0.101
Add salt before tasting the food (Often)	57 (14.2%)	30 (14.4%)	27 (14.1%)	0.679
Did you try to reduce salt intake before (Yes)	159 (39.7%)	76 (36.4%)	83 (43.2%)	0.272
Did you try to use spices to reduce salt (Yes)	154 (38.4%)	77 (36.8%)	77 (40.1%)	0.005
Which bottle water do you drink				
Regular filtered water	301 (75.1%)	167 (79.9%)	134 (69.8%)	0.062
Low sodium filtered water	73 (18.2%)	30 (14.4%)	43 (22.4%)	
I don’t know	27 (6.7%)	12 (5.7%)	15 (7.8%)	

^1^ Answer options for practice questions included often, sometimes, and never. Only answers of often are presented.

**Table 5:** Association of salt related knowledge, attitude, and practice scores among UOS students and their sociodemographic characteristics (*n* = 401).

Characteristics	Knowledge Score Out of 30 mean ± SD	Attitude Score Out of 5 OR [95% CI]	Practice Score Out of 13 OR [95% CI]
Age (years)			
18–20	16.4 ± 5.9	1.5 ± 1.3	4.5 ± 2.1
21–25	17.9 ± 6.1	1.4 ± 1.3	4.4 ± 2.3
*p*-value	0.010	0.550	0.797
Gender			
Male	15.9 ± 6.2	1.4 ± 1.2	4.3 ± 2.1
Female	18.5 ± 5.7	1.5 ± 1.3	4.7 ± 2.3
*p*-value	<0.001	0.377	0.116
Nationality			
Emirati	17.1 ± 5.4	1.4 ± 1.1	5.1 ± 2.5
Non-Emirati	17.2 ± 6.2	1.4 ± 1.3	4.4 ± 2.1
*p*-value	0.907	0.880	0.056
Health related major ^1^			
Yes	17.9 ± 5.8	1.5 ± 1.3	4.6 ± 2.3
No	16.3 ± 6.3	1.4 ± 1.1	4.3 ± 2.1
*p*-value	0.009	0.767	0.134
Residential type			
With Family	16.9 ± 6.2	1.5 ± 1.2	4.4 ± 2.1
Hostel	17.7 ± 5.7	1.3 ± 1.3	4.6 ± 2.3
Alone	18.2 ± 6.9	1.6 ± 1.2	5.5 ± 1.8
*p*-value	0.366	0.464	0.151
Marital status			
Single	17.1 ± 6.0	1.4 ± 1.3	4.5 ± 2.2
Married	18.1 ± 8.9	1.6 ± 1.5	4.9 ± 2.4
*p*-value	0.639	0.582	0.565
Most meal are consumed at			
Home	17.5 ± 5.8	1.7 ± 1.3	4.8 ± 2.3
Restaurants	16.6 ± 6.5	1.1 ± 1.1	3.9 ± 1.8
*p*-value	0.120	<0.001	<0.001
Body Mass Index			
Underweight	16.2 ± 3.7	1.3 ± 0.9	3.2 ± 0.8
Normal weight	17.4 ± 6.1	1.4 ± 1.2	4.4 ± 2.1
Overweight	16.5 ± 6.2	1.5 ± 1.3	4.4 ± 2.2
Obesity	17.8 ± 5.8	1.5 ± 1.4	4.5 ± 2.6
*p*-value	0.439	0.938	0.112
Blood Pressure			
Normal	17.6 ± 6.2	1.5 ± 1.3	4.6 ± 2.2
Elevated	16.5 ± 5.9	1.7 ± 1.2	4.2 ± 1.7
HBP ^2^ Stage 1	17.5 ± 5.6	1.3 ± 1.1	4.5 ± 2.2
HBP ^2^ Stage 2	16.3 ± 6.5	1.6 ± 1.4	4.4 ± 2.4
*p*-value	0.361	0.177	0.820

^1^ Health-related major includes health sciences, medicine, dental medicine, and pharmacy; ^2^ HBP: high blood pressure.

**Table 6:** Daily macronutrients and energy intake among UOS students (*n* = 122) by gender.

	Males (*n* = 56) mean ± SD	Females (*n* = 66) mean ± SD	*p*-Value
Energy			
Kcal/day	1970.7 ± 684.6	1741.4 ± 644.9	0.059
Proteins			
g/day	92.1 ± 41.0	72.1 ± 43.5	0.011
% energy	18.9 ± 7.6	16.6 ± 6.8	0.081
Carbohydrates			
g/day	212.2 ± 85.3	211.3 ± 78.0	0.955
% energy	43.7 ± 13.8	49.6 ± 10.3	0.007
Total fat			
g/day	86.5 ± 45.4	68.0 ± 35.8	0.013
% energy	37.5 ± 11.6	33.8 ± 9.1	0.052
Saturated fatty acid			
g/day	36.1 ± 29.7	25.7 ± 13.8	0.012
% energy	16.4 ± 14.7	13.1 ± 5.2	0.096
Monounsaturated fatty acids			
g/day	30.9 ± 18.2	21.5 ± 13.3	0.001
% energy	14.0 ± 6.5	10.7 ± 4.7	0.004
Polyunsaturated fatty acids (n-6)			
g/day	15.3 ± 10.0	10.5 ± 8.1	0.004
% energy	6.8 ± 3.5	5.2 ± 3.0	0.007
Trans fat			
g/day	2.6 ± 1.6	1.9 ± 1.4	0.024
% energy	1.1 ± 0.6	1.0 ± 0.5	0.113
Cholesterol (mg/day)	397.4 ± 243.1	210.2 ± 119.3	<0.001
Sodium (mg/day)	3677.4 ± 1209.6	3464.1 ± 1192.3	0.330
Potassium (mg/day)	1560.7 ± 350.4	1559.3 ± 463.0	0.985

**Table 7:** Percentage of UOS students not meeting the dietary recommendations (*n* = 122) by gender.

	Total (*n* = 122) *n* (%)	Males (*n* = 56) *n* (%)	Females (*n* = 66) *n* (%)	*p*-Value
<10% energy from protein	7 (5.7)	1 (1.8)	6 (9.1)	0.084
>35% energy from total fat	59 (48.4)	31 (55.4)	28 (42.4)	0.154
>7% energy from saturated fatty acid	110 (90.2)	51 (91.1)	59 (89.4)	0.757
>1% energy from trans fat	78 (63.9)	39 (69.6)	39 (59.1)	0.226
>300 mg/day cholesterol	35 (28.7)	28 (50)	7 (10.6)	< 0.001
>2300 mg/day sodium	109 (89.3)	50 (89.3)	59 (89.4)	0.985
<4700 mg/day potassium	122 (100)	56 (100)	66 (100)	- ^a^

^a^ No statistics were computed because (<4700 mg/day potassium) is a constant.
